# The Relevance of Assessing the Cell Proliferation Factor Ki-67 in Squamous Cell Carcinoma of the Larynx

**DOI:** 10.1155/2019/8142572

**Published:** 2019-01-15

**Authors:** J. Vukelic, R. Dobrila-Dintinjana, A. Dekanic, B. Marijic, A. Cubranic, T. Braut

**Affiliations:** ^1^Department for Otolaryngology and Head and Neck Surgery, Clinical Hospital Center Rijeka, Kresimirova 42, 51000 Rijeka, Croatia; ^2^Department for Radiotherapy and Oncology, Clinical Hospital Center Rijeka, Kresimirova 42, 51000 Rijeka, Croatia; ^3^Department of Pathology, Clinical Hospital Center Rijeka, Kresimirova 42, 51000 Rijeka, Croatia; ^4^Department for Internal Medicine, Clinical Hospital Center Rijeka, Kresimirova 42, 51000 Rijeka, Croatia

## Abstract

The aim of study was to investigate the expression of the proliferation factor Ki-67 and its relationship with histological grade, cancer stage, and treatment outcome in squamous cell carcinoma of the larynx. Samples from 78 patients with laryngeal cancer were analysed retrospectively. Paraffin sections of tumors were immunohistochemically stained for Ki-67 expression. The patients were divided in two groups according to the proliferative factor values (a low Ki-67 index group - Ki-67≤34 and high Ki-67 index group-Ki-67 >34). Statistical analysis of the data shows significant correlation among histological tumor grade and the value of the Ki-67 proliferative index. There was no correlation between tumoral Ki-67 expression and diagnosis, stage of the disease, or treatment outcome. In conclusion, Ki-67 expression in laryngeal cancer is not the most reliable marker for making precise diagnosis and predicting the clinical course.

## 1. Introduction

Malignant neoplasms of the larynx are the most common malignancies of the upper respiratory tract in adults [1]. The biggest treatment challenge is to predict the course of the disease and develop a treatment plan accordingly. Great efforts have been made in order to determine the relevance of potential prognostic factors, including clinical stage, site and size of the tumor, histological grade, and depth of tumor invasion. To date, however, none of the parameters have either proven to be reliable or to have any irrefutable clinical value as a prognostic factor [2]. Treatment planning relies on histopathological diagnosis and assessment of the tumor-nodus-metastasis (TNM) stage. A precise histolopathological grading of neoplasms is extremely important for a reliable prognosis but we must be aware of nonstandardized criteria resulting in poor precision of this tool [3]. On the other hand, TNM provides prognostic information that guides treatment for cohorts of patients with laryngeal cancer but is limited by its inability to predict success on an individual basis [4].

An important feature of malignant transformation is the deregulation of the cell cycle as shown by a large number and high proliferation rate of dividing cells. In a number of studies, several approaches have been adopted to determine the proliferative activity of tumor tissue as accurately as possible. The measurement has included the determination of the S-phase fractions, mitotic index, and histone H3 mRNA [5–7]. Most of the studies have produced contradictory results on proliferative activity and disease prognosis [8]. More reliable results have been obtained using Ki-67 as a proliferation marker [6, 9].

Ki-67 is a nuclear protein that is responsible for cell proliferation. It is only expressed in the cell division cycle: interphase (G1, S, and G2), prophase, metaphase, anaphase, and telophase. Its expression is absent in the G0 phase,and, therefore, Ki-67 is an excellent marker of cell division [10].

The results of the study by De Vincentiis and Bai demonstrated increased expression of the Ki-67 proliferative index in both patients with poorly differentiated carcinomas and patients with regional lymph node metastases [11, 12]. In contrast to the above, these results were not confirmed by other authors [13, 14]. Ki-67 expression correlates positively with tumor grade and pathological stage of the disease [2, 14]. Acikalin et al. found a significant correlation between Ki-67 expression and patient age and tumor stage and lymph node metastases [15]. A study of a cohort of 4,806 patients demonstrated a correlation between Ki-67 expression and disease prognosis [6]. Gioacchini et al. performed a systematic review on articles dealing with Ki-67 immunohistochemical staining and laryngeal squamous cell carcinoma. Their review strongly suggests that immunohistochemical staining of Ki-67 correlates with tumoral aggressiveness and worse prognosis in patients [16]. Ki-67 can be used as an important indicator for judging clinical progress and estimating prognosis in human laryngeal squamous carcinoma [12].

The aim of this study is to investigate the expression of the proliferation factor Ki-67 and its relationship with histological grade, cancer stage, and treatment outcome in squamous cell carcinoma of the larynx. This will enable us to develop our own opinion on benefits of the proliferation factor Ki-67 in diagnosis, treatment, and follow-up of patients with malignant tumors of the larynx.

## 2. Materials and Methods

### 2.1. Materials

A retrospective study was performed on 78 tissue samples from patients with laryngeal cancer. The study included patients diagnosed with squamous cell carcinoma of the larynx. The patients were divided into two groups. The first group consisted of 68 (87.2%) patients diagnosed with laryngeal cancer, and the second group included 10 (12.8%) patients with laryngeal cancer and regional lymph node metastases. The patients' age at diagnosis ranged from 45 to 81 years, with the median of 60 years. All the patients underwent surgical treatment. A partial laryngectomy was performed in 36, and 42 patients were submitted to a total laryngectomy. A total of 55 patients underwent oncology treatment: radiation therapy alone was administered to 47 and chemoradiotherapy to 8 patients. Patients were further divided according to the stage of their disease. As literature suggests, stages 1 and 2 were considered early stage, and stages 3 and 4 were classified as advanced stage [17]. There were 36 (46.2%) and 42 (53.8%) patients in the early and advanced stage of the disease, respectively. As regards histological grade, the patients were divided into four groups. Patients with histological grade 2 (51.3%) accounted for the largest share, followed by patients with grade 1 (23.1%) and then those with grade 3 (14.1%), whereas the share of grade 0 tumor patients was the smallest (11.5%). For all patients, information on local recurrence, regional lymph node metastases, and occurrence of a second primary tumor was collected from their medical records. Local recurrence was reported in 8 (10.2%) patients, regional lymph node metastases were reported in 9 (11.5%), and a second primary tumor was found in 7 (9.0%) patients. The patients were followed up for 5 years after surgery. The total number of patients alive after 5 years was 50 (64.1%). Given that this was a bench research study, there was no need to establish a control group.

### 2.2. Methods

Tissue samples of invasive and* in situ* squamous cell carcinoma were stained by immunohistochemistry. Histopathological analysis was performed using light microscopy. All the specimens were fixed in 10% buffered formalin (Kemika, Zagreb, Croatia), embedded into paraffin blocks, and stained with hematoxylin and eosin (HE). The specimens were determined for the degree of histologic differentiation according to the World Health Organization grading scheme.

### 2.3. Immunohistochemical Analysis

Representative tissue samples of laryngeal carcinoma were selected to make a paraffin block further cut into into 4-5*μ*m thick sections. Overnight, sections were kept in a thermostat at 37°C, then deparaffinized using the standard procedure with a xylene substitute (Tissue Clear Sakura) (3x10′), and rehydrated in absolute ethyl alcohol (2x5′), 96% ethyl alcohol (5′), and 70% ethyl alcohol (5′) until washed with distilled water. The samples were treated using the EnVision method-based visualization system. The visualization system (Dako Real EnVision Detection System Peroxidase/DAB+, Rabbit/Mouse K5007) was used with an automated immunostainer (Dako Autostainer Plus). Diaminobenzidine (DAB) was employed as chromogen. The primary antibody used was Ki-67 Antigen Clone MIB-1 M7240. After implementation of the visualization system, the sections were counterstained with hematoxylin for 1 minute, washed with tepid water, dehydrated in ethyl alcohol of different concentration (70%, 96% and 100%), and then rinsed in a xylene substitute. Finally, coverslips were mounted in GLC Mounting Medium. Washes between each step were performed using Dako Wash Buffer 10x S 3006.

### 2.4. Evaluation of Proliferative Index

The evaluation of Ki-67 expression on tumor cells was performed using present information provided in the available literature. The expression was evaluated two independent pathologists N.J. and A.D. Ki-67 expression was counted as a percentage. The evaluation was carried out using the following procedure: quantification of structures was performed by the Alphelys Spot Browser 2 integrated system consisting of a software-controlled (Alphelys Spot Browser 2.4.4) motorized microscope (Nikon Eclipse 50i) with a mounted digital camera (Microvision CFW-1310C, 24 bits, 1360x1024 pixel resolution).

The system was calibrated by the Nazca software (Microvision Instruments, France) which determines the size of a pixel for each lens (0.3311 *μ*m for 20x lens and 3.320 *μ*m for 2x).

After having taken overview images of the slides at 20x magnification, images of the selected areas were taken at 200x magnification to be analyzed with image analysis software tools, under constant adjustments of camera and microscope settings.

Digital image analysis detects objects based on color (wavelength, intensity, and saturation), groupings and morphological features (size and shape). Objects are marked with colors, which enables a control and correction of the detection process.

For this purpose, a detection algorithm that specifies conditions for detection of objects of interest quantification has been developed.

During the detection process, the software automatically performs the measurement of detected objects and calculation of default parameters, in this case the number of positive and negative tumor cell nuclei.

### 2.5. Statistical Analysis

Statistical analysis of the collected data was performed using Statistica® software package (Version 10.0, StatSoft Inc. Tulsa, OK, USA). Appropriate statistical tests were employed to analyze the collected data, with a statistical significance level of p<0.05 to form a conclusion.

## 3. Results

The patients were divided into two groups according to Ki-67 proliferative index values: a low Ki-67 index group (Ki-67≤34) and high Ki-67 index group (Ki-67 >34).

Table 1 shows the frequency of low and high Ki-67 proliferative index in relation to histological grade. Statistical analysis of the data contained in the table reveals a statistically significant correlation between histological tumor grade and the value of the Ki-67 proliferative index (Pearson's *χ*^2^ test, *χ*^2^ =9.22, and p=0.026), with significant departures from the expected frequencies for histological grades 2 and 3. In grade 2 tumors, patients with the proliferative index >34 significantly prevail, whereas in grade 3 patients, there is a significant prevalence of the proliferative index ≤34.

Table 2 shows patient group-adjusted proliferative index values in relation to diagnosis. Analysis of the data contained in the table indicates that there is no correlation between diagnosis and proliferative index (Pearson's *χ*^2^ test, *χ*^2^ =0.60, p=0.806).

Table 3 displays the patient group-adjusted frequency of grouped proliferative index values in relation to the stage of the disease. Analysis of the data contained in this table reveals no correlation between the two sets of data (Pearson's *χ*^2^ test, *χ*^2^ =0.63, p=0.438).

Table 4 shows the patient group-adjusted frequency of grouped proliferative index values with respect to treatment outcome. Analysis of the data contained in this table demonstrates that no correlation exists between the two sets of data (Pearson's *χ*^2^ test, *χ*^2^ =0.14, p=0.705).

## 4. Discussion

Cell proliferation is among the most important factors involved in the biological process of oncogenesis. Over the last few years, there has been a significant progress in the development of a number of antibodies associated with proliferation factors. In laryngeal carcinoma, Ki-67 is the most widely used factor of cell proliferation. There is no clear cut-off that can be used to determine low or high proliferation rates. In our study, we have used the mean value calculated according to the available literature data [8, 10, 18–20]. Patients with Ki-67 levels under and equal to 34% were assigned to the low Ki-67 group, whereas the remaining ones were assigned to the high Ki-67 group.

In our study, we observed the relationship between the histological grade and expression of the proliferation factor Ki-67. A majority of studies to date advocate a positive correlation between the expression of the Ki-67 proliferative index and histological grade. Mondal et al. found a significant correlation between Ki-67 expression and histological grade [21]. Cui et al. performed a retrospective analysis of a large sample size of 555 cases of vocal cord leukoplakia, consisted of keratosis, hyperplasia, dysplasia, and malignancies. The expression of the proliferation factor Ki-67 was significantly associated with histopatological grade. All of the malignant cases in this patient cohort were found positive in Ki67 staining [22]. Pastuszewski et al. also provided evidence of an increased expression of Ki-67 related to an increase in histological grade, with the highest levels having been detected in histological grade 3 [23]. Sarafoleanu et al. confirmed a statistically significant positive correlation between the expression of the proliferation factor Ki-67 and histological grade [24]. Our study produced unexpected results as, based on current knowledge, the assumption had been that the proliferation index would increase with an increase in histological grade. In our study, uncorroborated positive correlation between a higher histological grade and a higher Ki-67 level further suggests the relevance of both the Ki-67 proliferative index assessment and histological grading. However, histological grading is a subjective method, and this quality is considered to be its disadvantage. Ki-67 proliferation factor values should therefore be investigated along with the proliferative index and other parameters in order to, taking into account the overall results, draw conclusions on the clinical course and/or treatment.

Previous studies on the proliferative index in relation to the clinical course and treatment outcome have produced markedly contradictory results. The study by Cordes et al. demonstrated a 5-year-survival rate of 84% for patients with low proliferative index, whereas the survival rate for patients with high proliferative index was 47.18% [8]. In contrast to the above, the study by Teppo et al reported that Ki-67 did not significantly affect the prognosis of laryngeal cancer [25]. Research conducted on 2077 patients demonstrated a correlation between high proliferative index and poor prognosis, whereas another one that included 1,212 patients refuted the results of the previous study [6].

Our study did not provide evidence of any correlation between proliferative index and either stage of the disease or treatment outcome, which to some extent complies with published results. Pastuszewski et al. also did not find any relationship between the expression of Ki-67 proliferative index and stage of the disease, but demonstrated a significantly shortened survival in patients with a high expression of Ki-67 [23]. In a study conducted on a cohort of 108 patients with malignant neoplasms of the larynx, Re et al. showed a significantly lower expression of Ki-67 in the early stage of the disease as compared to the advanced stage [26].

With a view to the above results, we may conclude that neither Ki-67 nor its role in the treatment of patients with malignant neoplasms of the larynx has been precisely defined. Further studies are needed to clarify the role of Ki-67 and enable definite conclusions about its utility in everyday clinical practice.

## 5. Conclusion

Precise diagnosis and prediction of the clinical course in patients with malignant neoplasms of the larynx represent a great challenge. Great efforts are made to identify an ideal proliferation marker that might indicate the clinical course and treatment outcome of patients with malignant neoplasms. The general properties of such a marker should include simple detection, unambiguous results as regards the outcome and clinical course of the disease, and affordability. The use of such a reliable proliferation marker would enable modification of the current guidelines in order to ensure more accurate patient treatment and avoid both undertreatment or overtreatment and excessive diagnostic testing. As present results related to Ki-67 have not yet been substantiated enough and have as well been contradictory, we may conclude that additional investigations are required to more precisely define Ki-67 and its role in the treatment of patients with malignant neoplasms of the larynx.

## Figures and Tables

**Table 1:** Patient group-adjusted frequency of grouped proliferative index values and histological tumor grade.

	**Tumor proliferative index**		
**Histological tumor grade**	**Low (≤34)**	**High (>34)**	**Total**
**0**	5	4	9

**1**	7	11	18

**2**	13	27	40

**3**	9	2	11

**Total**	34	44	78

**Table 2:** Patient group-adjusted frequency of grouped proliferative index values by diagnosis.

	**Tumor proliferative index**		
**Diagnosis**	**Low (≤34)**	**High (>34)**	**Total**
**Malignant laryngeal neoplasm**	30	38	68

**Malignant laryngeal neoplasm+ regional lymph node metastases**	4	6	10

**Total**	34	44	78

**Table 3:** Patient group-adjusted frequency of grouped proliferative index values and the stage of the disease.

	**Tumor proliferative index**		
**Stage**	**Low (≤34)**	**High (>34)**	**Total**
Early	14	22	36

Advanced	20	22	42

Total	34	44	78

**Table 4:** Patient group-adjusted frequency of grouped proliferative index values and treatment outcome.

	**Tumor proliferative index**		
**Treatment outcome**	**Low (≤34)**	**High (>34)**	**Total**
**Alive**	21	29	50

**Deceased**	13	15	28

**Total**	34	44	78

## Data Availability

The data about tissue samples and data from patients' medical history used to support the findings of this study are restricted by the Ethics board of Clinical Hospital Center Rijeka in order to protect patient privacy. Data are available from Jelena Vukelic (jl.vukelic@gmail.com), for researchers who meet the criteria for access to confidential data.
